# Electrospinning of Block and Graft Type Silicone Modified Polyurethane Nanofibers

**DOI:** 10.3390/nano9010034

**Published:** 2018-12-27

**Authors:** Chuan Yin, Rino Okamoto, Mikihisa Kondo, Toshihisa Tanaka, Hatsuhiko Hattori, Masaki Tanaka, Hiromasa Sato, Shota Iino, Yoshitaka Koshiro

**Affiliations:** 1 Interdisciplinary Graduate School of Science and Technology, Shinshu University, 3-15-1, Tokida, Ueda-shi, Nagano 386-8567, Japan; yinkawa@outlook.com (C.Y.); j3992a1953m@gmail.com (R.O.); Kondo.mikihisa@exc.epson.co.jp (M.K.);; 2 Silicone-Electronics Materials Research Center, Shin-Etsu Chemical Co., 1-10, Hitomi, Matsuida-Machi, Annaka-Shi, Gunma 379-0224, Japan; hhattori@shinetsu.jp (H.H.); m.tanaka@shinetsu.jp (M.T.); 3 Dainichiseika Color & Chemicals Mfg. Co., 1-4-3, Ukima, Kita-ku, Tokyo 115-8622, Japan.; hrsato@daicolor.co.jp (H.S.); iino@daicolor.co.jp (S.I.); koshiro@daicolor.co.jp (Y.K.)

**Keywords:** Silicone modified polyurethane, electrospun nanofibers, optimization, upscaling

## Abstract

Silicone modified polyurethane (PUSX) has attracted interest as a useful material by various properties, which are combined with silicone and polyurethane. In this paper, we tried to optimize the electrospinning process of silicone modified polyurethane (PUSX) nanofibers on a lab scale device and a multinozzle pilot scale set-up to investigate the potential and limitations of preparing PUSX nanofibrous sheets using different equipment. The morphology and diameter of the obtained fibers were studied via scanning electron microscopy (SEM). Attenuated total reflectance-Fourier transform infrared spectroscopy (ATR-FTIR) was also carried out to analyze the chemical structure of PUSX nanofibers. As a result, we successfully figured out the optimal parameters of PUSX electrospinning process and demonstrated the great potential of the process for mass production of PUSX nanofibrous sheets from solutions.

## 1. Introduction

Silicones are highly functional resins that combine the characteristics of both inorganic and organic substances and exhibit an array of useful properties, including heat resistance, cold resistance, weather ability, dielectric properties, release properties, and water repellency. By introducing silicones into other resins, such as polyamide, polyimide, polyester, polycarbonate, and polyurethane, new resin materials can be created with enhanced functionality that include not only heat resistance, weather ability, and flame resistance, but improved impact resistance, lubricity, and flexibility. With their enhanced functionality, these high-performance composite resins are lightweight and have excellent fabricating characteristics and are used in a myriad of fields, including electric and electronics applications, automotive applications, electrical wire, and construction [1]. For instance, silicone-modified acrylic emulsions with hydroxyl groups may be used as automotive coatings, wood finishes, maintenance, and plastic coatings [2].

Polyurethane (PU) is formed by reaction of polyisocyanates with hydroxyl-containing compounds. Desired properties can be tailored by selecting the type of isocyanate and polyols, or combination of isocyanates and polyols [3]. Strong intermolecular bonds make polyurethanes useful for diverse applications in adhesives and coatings, also in elastomers, foams, and medical applications because of their good flexibility. Nevertheless, there are still a lot of disadvantages and limitations of polyurethane materials, such as poor thermal capability, poor weatherability, and flammability. In order to apply PU in more fields, many efforts were made by combining PU and silicone together to improve those properties and break through the limitations. Leonard D. Tijing et al. suggested a way to combine the advantages of silicone film and PU nanofibers containing carbon tubes to improve the mechanical properties and got the nanofibers well-embedded in the silicone film matrix, but the existence of CNT shows higher cost and lower flexibility [4]. Chan-Hee Park et al. successfully prepared the (polyurethane/nylon-6) nanofiber/ (silicone) film composites via electrospinning and dip-coating to get an obvious increase in tensile strength [5]

Here, we tried to get the advantages of both PU and silicone by introducing silicone groups into polyurethane polymer chains. Silicone modified polyurethane (PUSX) has attracted as useful material by various properties, which combined with silicone and polyurethane, such as heat resistance, weather resistance, peeling of molding, oil resistance, and high mechanical strength [6]. As one of the similar examples of our material, PurSil™ thermoplastic silicone polyether urethane has been proven with biocompatibility and biostability, containing silicone as a soft segment, which is prepared by incorporating polydimethylsiloxane into the polymer soft segment with polytetramethyleneoxide and the hard segment consists of an aromatic diisocyanate [7]. In our research, we synthesized the copolymer into different structures containing different segments compared with PurSil™. Currently, we use PUSX as films, molding products, or adhesive, but there is no report of PUSX as a fiber, specifically as a nanofiber. Electrospun nanofibers, including polyurethane nanofibers, have attracted much attention due to their unique properties, such as a very large surface area to volume ratio, flexibility in surface functionalities, superior mechanical performance, high porosity, and good interfacial adhesion [8,9]. PU nanofibers have been perfectly prepared and studied by a large number of researchers in recent years. For instance, Haitao Zhuo et al. discussed the influence of electrospinning parameters, including the applied voltage, feeding rate, and solution concentration, on the diameters and morphology of PU nanofibers [10] while F. Cengiz et al. investigated the effect of tetraethylammoniumbromide salt on the spinnability of PU nanofibers [11]. S. Thandavamoorthy et al. discovered the self-assembling phenomenon in electrospun PU nanofibers [12]. However, PUSX nanofibers are not that easy to prepare because of the existence of silicone. To make silicone into electrospun nanofibers, several ways have been reported by researchers. Haitao Niu et al. suggested a modified core–shell electrospinning method using a commercially available liquid polydimethylsiloxane (PDMS) precursor and polyvinylpyrrolidone (PVP) as core and sheath materials to prepare continuous ultrafine PDMS fibers, with an average diameter of around 1.35 μm [13]. After removing PVP by the dissolving method, the PDMS fiber surface was not as ideal as expected. Ruiping Xue et al. also tried the core-shell electrospinning method to prepare PDMS core-polycaprolactone (PCL) shell nanofibers for biocompatible, real-time oxygen sensor applications [14]. Since the PDMS core was very viscous, the existence of thin PCL shell became essential to maintain the morphology. Both of them showed some limitations of the core-shell structure silicone nanofibers. Miriam Haerst et al. showed a fast crosslink way to prepare PDMS nanofibers by electrospinning PDMS-acetone solutions on a 100 °C heat plate. Unfortunately, the average diameter appeared to be large compared to other electrospun polymer nanofibers [15]. On the other hand, nanofibers made from silicone resins have been reported [16], organopolysiloxane fibers with diameters ranging from 0.01µm to 100µm were prepared by an electrospinning process. Also, the electrospun silsesquioxane nanofibers for battery separator materials were reported [17] Unfortunately, both of the reports could not show the uniform nanofibers with ideal fine diameters, thus the physical properties and other characteristics are still unknown.

In this research, we tried to synthesize the silicone modified polyurethane (PUSX) copolymers instead of using blends of two polymers and we optimized the electrospinning process of PUSX nanofibers on a lab scale device and a multinozzle pilot scale set-up to investigate the potential and limitations of preparing PUSX nanofibrous sheets using different equipment. The morphology and diameter of the obtained fibers were studied via scanning electron microscopy (SEM). Attenuated total reflectance-Fourier transform infrared spectroscopy (ATR-FTIR) was also carried out to analyze the chemical structure of PUSX nanofibers.

## 2. Materials and Methods

### 2.1. Materials

**Scheme 1:**
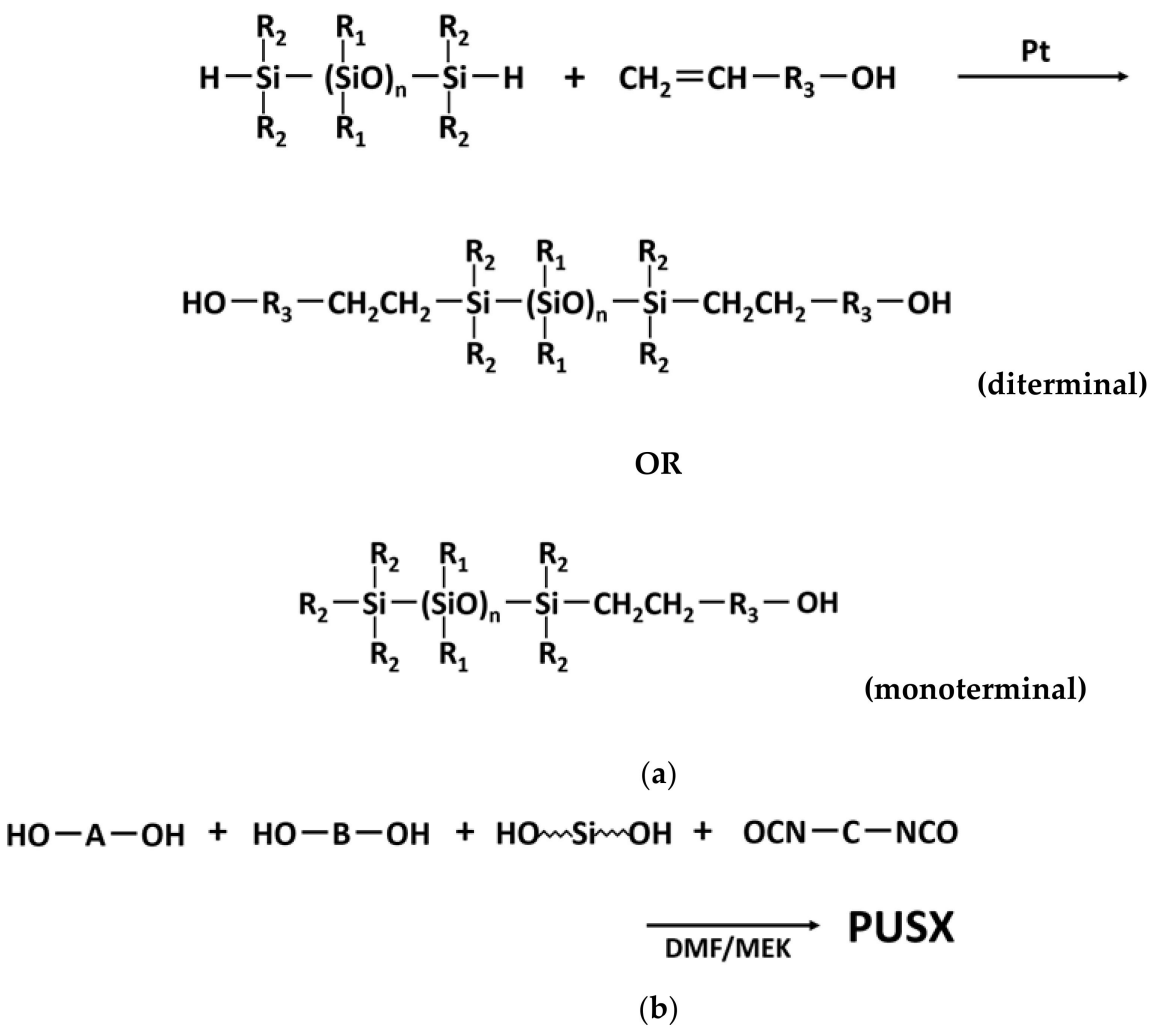
(**a**) Synthesis method of carbinol-modified silicone (silicone polyol); (**b**) Synthesis method of PUSX.

All the 12 kinds of silicone modified polyurethane (PUSX) solutions were kindly synthesized and provided by Shin-Etsu Chemical Co., Ltd (Tokyo, Japan) and Dainichiseika Color & Chemicals Mfg. Co., Ltd (Tokyo, Japan). The weight average molecular weight (Mw) is a polymethyl methacrylate (PMMA) equivalent value measured by gel permeation chromatography (GPC). GPC measurements were carried out with an HLC-8320 GPC system (Tosoh Corporation, Tokyo, Japan), tetrahydrofuran (THF) as the solvent, and at a resin concentration of 0.1%. Defined PUSX with Mw ranging from 1.48×10^5^ to 2.33 × 10^5^ were synthesized as described in Scheme 1. Solvents, such as N,N-dimethylformamide (DMF) and ethyl methyl ketone (MEK), were purchased from Wako Pure Chemical Industries., Ltd (Osaka, Japan) and used as received.

### 2.2. Synthesis of PUSX

The carbinol-modified silicone (silicone polyol) used in the synthesis process of PUSX was hydrosilylated by organopolysiloxane (with Si-H group) and unsaturated alcohol compound, catalyzed by platinum catalyst. The synthesis of block type silicone polyol (diterminal diols) is shown in Scheme 1a, graft type silicone polyol (monoterminal diols) can also be prepared by this method. Here, both R_1_ and R_2_ are methyl group (-CH_3_) while R_3_ is a -OC_2_H_4_- group.

**Figure 1:**
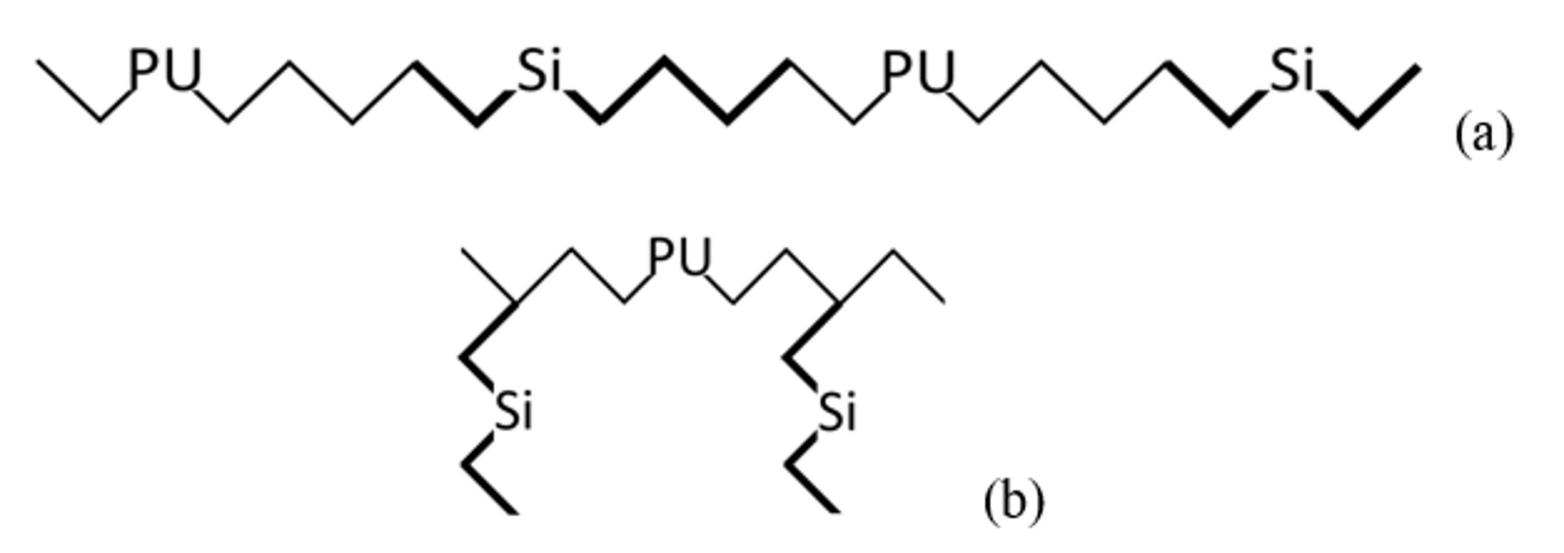
Structures of block type PUSX (**a**) and graft type PUSX (**b**).

Polytetramethylene glycol (polyol component: A in Scheme 1b) and 1,4-butanediol (chain extender: B in Scheme 1b) were used to form the polyurethane resin as a common component. To synthesize PUSX, it is essential to add silicone polyol copolymer structures (block type and graft type) and different silicone chain lengths (the number of repeat units of dimethylsiloxane: n), followed by specific reaction with the diisocyanate component in Scheme 1b in DMF/MEK mixed solvent. These synthesized PUSX solutions in DMF/MEK standardized a solid content of 30wt %. The structures of two types of PUSX are shown in Figure 1. Synthesized PUSX with different silicone concentrations and chain lengths are listed in Table 1.

### 2.3. Electrospinning Devices

**Figure 2:**
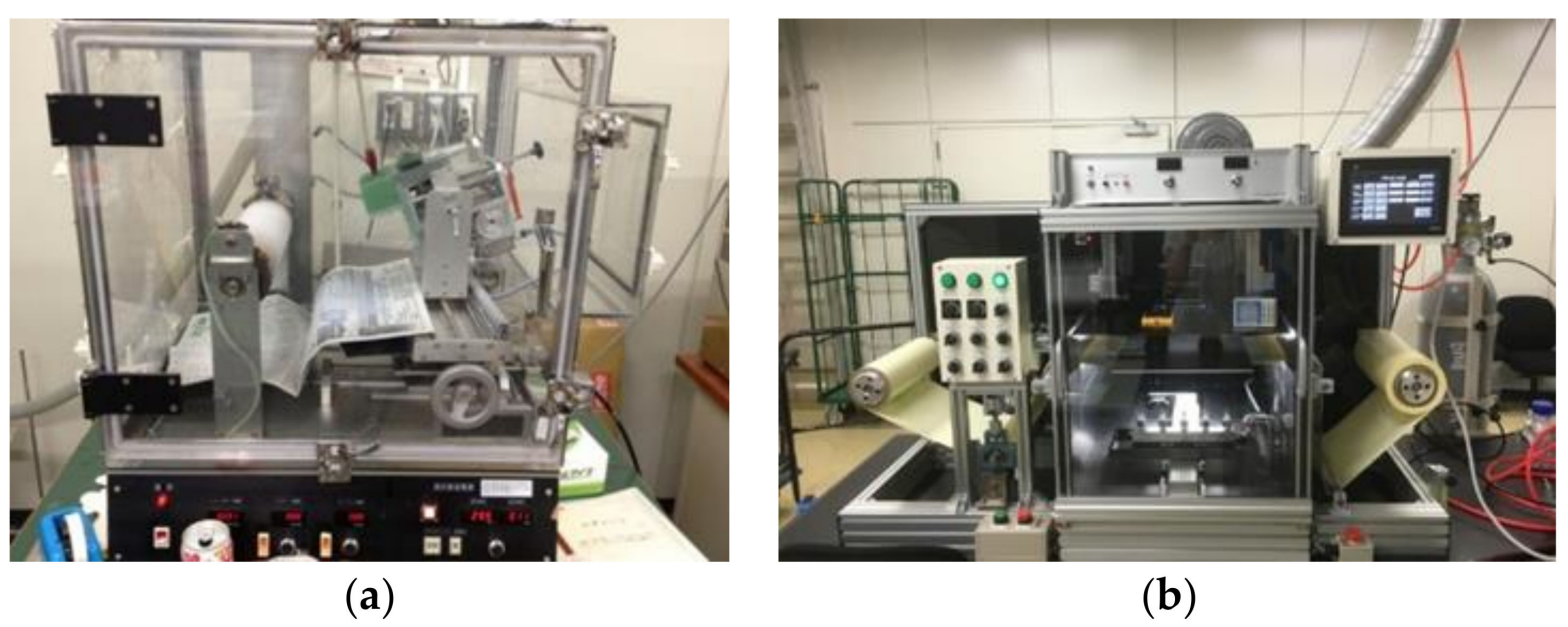
The lab scale electrospinning set-up (**a**) and pilot scale electrospinning set-up (**b**).

Two kinds of set-ups of the electrospinning devices were applied for the production of PUSX nanofibers. To explore the electrospinnabiliy of PUSX, a horizontal mononozzle lab scale device (as shown in Figure 2a) (NEU Nanofiber Electrospinning Unit, Kato Tech Co., Ltd, Kyoto, Japan) was used. Tips of metal injection needles (ø0.6 mm, length 26 mm) were cut before being used and placed 30° to the floor. According to Oliver Hardick et al’s study, the atmospheric conditions most suitable for cellulose acetate nanofiber production are 25.0 °C and 50% RH, which gives the highest level of fiber diameter uniformity, the lowest level of beading, and maintains a low fiber diameter for increased surface area and increased pore size homogeneity [18]. Moreover, Ji Zhou et al suggested that for PU nanofibers, the Young’s modulus decreases linearly with the increase in temperature in the range of 25–60 °C [19]. In this experiment, the environment conditions, such as humidity and temperature, have been already optimized and determined to be 50% RH and 22 °C controlled by a lab scale air compressor, an adsorption air dryer, and a micro flow rate temperature and humidity control apparatus. The data of temperature and humidity optimization is not shown in this paper.

Additionally, to verify the reproducibility of the process, a pilot scale vertical electrospinning set-up (as shown in Figure 2b) (Nafias ES300, NafiaS Inc., Nagano, Japan) was utilized. Five tips of plastic injection needles (ø0.70 mm, length 38 mm) were used and placed vertical to the floor. The collector was settled above the tips. The humidity was 50% RH and the spinning temperature was 22 °C under the control of a small-sized air compressor and micro flow rate temperature and humidity control apparatus.

### 2.4. Electrospinning Parameters 

Various electrospinning solutions were prepared by diluting the PUSX solutions (30 wt %, which is the most suitable concentration for synthesis) in DMF/MEK mixed solvent and stirring at room temperature for 48 h to obtain homogeneous solutions. To investigate the optimized parameters, all electrospinning experiments were performed by a mononozzle lab scale device at room temperature (22 °C) and the deposited nanofibers were collected on a drum shape rotating metallic collector. A 10–20 kV voltage was applied while the needle tip to collector distance was 10 cm with the irradiation angle of 30° and air flow rate in the spinning environment was 0.1 mL/min. The detailed relevant electrospinning parameters applied for each sample are discussed in Section 3.1.

### 2.5. Characterization of Applied Polymer Solutions and Prepared Nanofibers

#### 2.5.1. Viscosity Measurements

The apparent viscosity of the polymer solutions with different concentrations was measured using a digital rotational viscometer (Toki Sangyo Co., Ltd. TVB-10M, Tokyo, Japan). Software was used for the complete external control of the viscometer. Before the measurements, the viscometer was calibrated with standard oil (1000 mPa*s and 10,000 mPa*s, standardized by Cannon-Fenske Viscometer), and the computed maximum uncertainty in the viscosity measurement was lower than 5%.

The measurement of the surface tension of the solution was performed by a simple surface and interfacial tensiometer (Kyowa Interface Science Co.,Ltd. DY-500, Saitama, Japan). The Wilhelmy plate method was employed. It is similar to the du Noüy ring method, but it is simpler and does not require correction. In this method, the plate is oriented perpendicular to the interface, and the force exerted on it is measured. This instrument was computer controlled, and it was calibrated with a known weight of 400 mg. The results we collected were the average of more than three specimens of each sample, with the computed maximum uncertainty lower than 5%.

For both viscosity and surface tension measurement, the number of replicates tested for each solution was three.

#### 2.5.2. Characterization

The surface morphology of nanofibers was investigated by scanning electron microscope (SEM, JSM-6010LA JEOL, Tokyo, Japan) at an accelerating voltage of 10 kV. The prepared sample before SEM analysis was coated using a platinum sputter coater (Ion sputter JFC-1600 JEOL Ltd, Tokyo, Japan) under 30 mA and 30 seconds to be observed by SEM. The diameters of nanofibers were measured by Image J (National Institutes of Health, Bethesda, Maryland (MD). US). The average fiber diameters and their standard deviations were calculated from data of at least 50 measurements per sample. At least three specimens were observed for each sample.

Attenuated total reflectance-Fourier transform infrared spectroscopy (ATR-FTIR, IR Prestige-21, Shimadzu Corporation, Kyoto, Japan) was carried out to analyze the chemical structure of PUSX nanofibers. All spectra were taken in an absorption mode between the wavenumber range of 4000–700 cm^−1^ with a resolution of 4 cm^−1^ and accumulation of 20 scans. The number of specimens observed for each polymer was three.

## 3. Results and Discussions

### 3.1. Electrospinning of PUSX Solutions (Optimizations of Electrospinning Process)

The electrospinning process is influenced by many parameters that consecutively also affect the morphology and the diameter of the obtained fibers. These parameters can be subdivided into polymer related and processing parameters. The predominant polymer related parameters, including the molecular weight, polymer concentration, and the solution viscosity, were varied as shown in Table 1. From Table 1, we can see that the surface tension decreased with the increase of both the silicone chain length and silicone concentration while the viscosity decreased only with the increase of silicone concentration in block type PUSX materials. Furthermore, the processing parameters, such as the applied voltage, the flow rate, and the needle to collector distance, were adjusted to optimize the electrospinning process.

In the first part of this work, the influence of the polymer concentration and applied voltage, viscosity on the fiber diameter was studied. Then, the optimized electrospinning parameters were transferred to a multinozzle pilot scale set-up to investigate the reproducibility of the fiber production method, the transferability of the electrospinning parameters, and the feasibility of upscaling the procedure.

#### 3.1.1. Effect of Polymer Concentration

Solution concentration is one of the most significant parameters for electrospinning and affects the viscosity. Electrospinning of polymer solutions with too low concentrations results in the formation of beads and heterogeneous fibers, and upon increasing the concentration, their shape evolves from spheres to spindles until uniform fibers are produced at the appropriate concentration. Conversely, high concentration leads to too viscous solutions for which continuous flow cannot be maintained, leading to electrospinning instability and the formation of thick and inhomogeneous fibers. Thus, an optimal concentration range for the electrospinning of polymers, resulting in stable and reproducible fiber production, must be determined. The optimal range for successful electrospinning of all the different PUSX fibers was around 10 wt %~20 wt % according to Table 2. 

Table 2 shows the SEM morphologies of electrospun block type and graft type PUSX fibers with a solution concentrations’ range of 0~10 wt %, 10~15 wt %, and 15~20 wt %. At higher concentrations, the presence of PUSX polymer is much more than that at lower concentration, resulting in enhanced interaction and entanglement of chains occurred to resist deformation. For Si01 and Si02 nanofibers, the amount of beads decreased with increasing polymer concentration, and eventually became uniform fibers. Irregular beads were formed because the very low viscosity did not suffice to sustain the elongation of the liquid jet, and therefore the thin jet of solution left the nozzle instantly and shrunk to droplets. For Si03, Si04, Si01-20, Si01-40, and Si01-59 nanofibers, low concentration led to irregular beads, but too high a concentration also led to too viscous solutions for which continuous flow could not be maintained. Especially, for Si01-40 and Si01-59, uniform and continuous nanofibers could not be formed when the polymer concentration was higher than 10 wt % because the ratio of silicone to PU was much higher in Si01-40 and Si01-59 than in other block type PUSX materials. Because of the electric insulting effect of silicone groups, as the polymer concentration increased, the electrostatic repulsion became more and more difficult, which explained the difficulties of the electrospinning process. In this case, the higher polymer concentration led to larger diameter of nanofibers and a shorter spinning time of less than 30 min (* in Table 2). For graft type PUSX nanofibers, the silicone groups on the side chains could not influence the structure too much which, made the spinability very similar to PU nanofibers.

#### 3.1.2. Effect of Different Solvent Ratio

**Figure 3:**
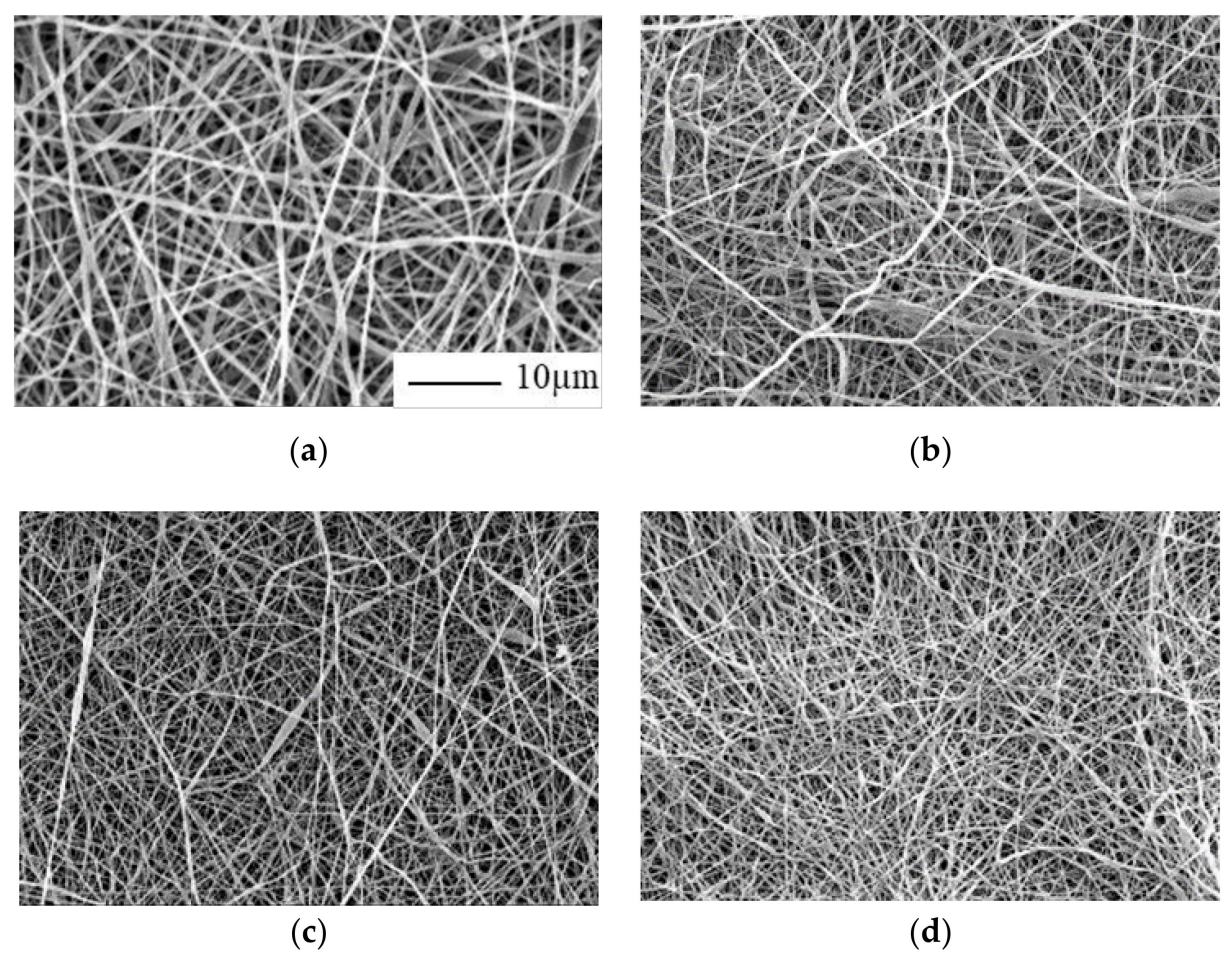

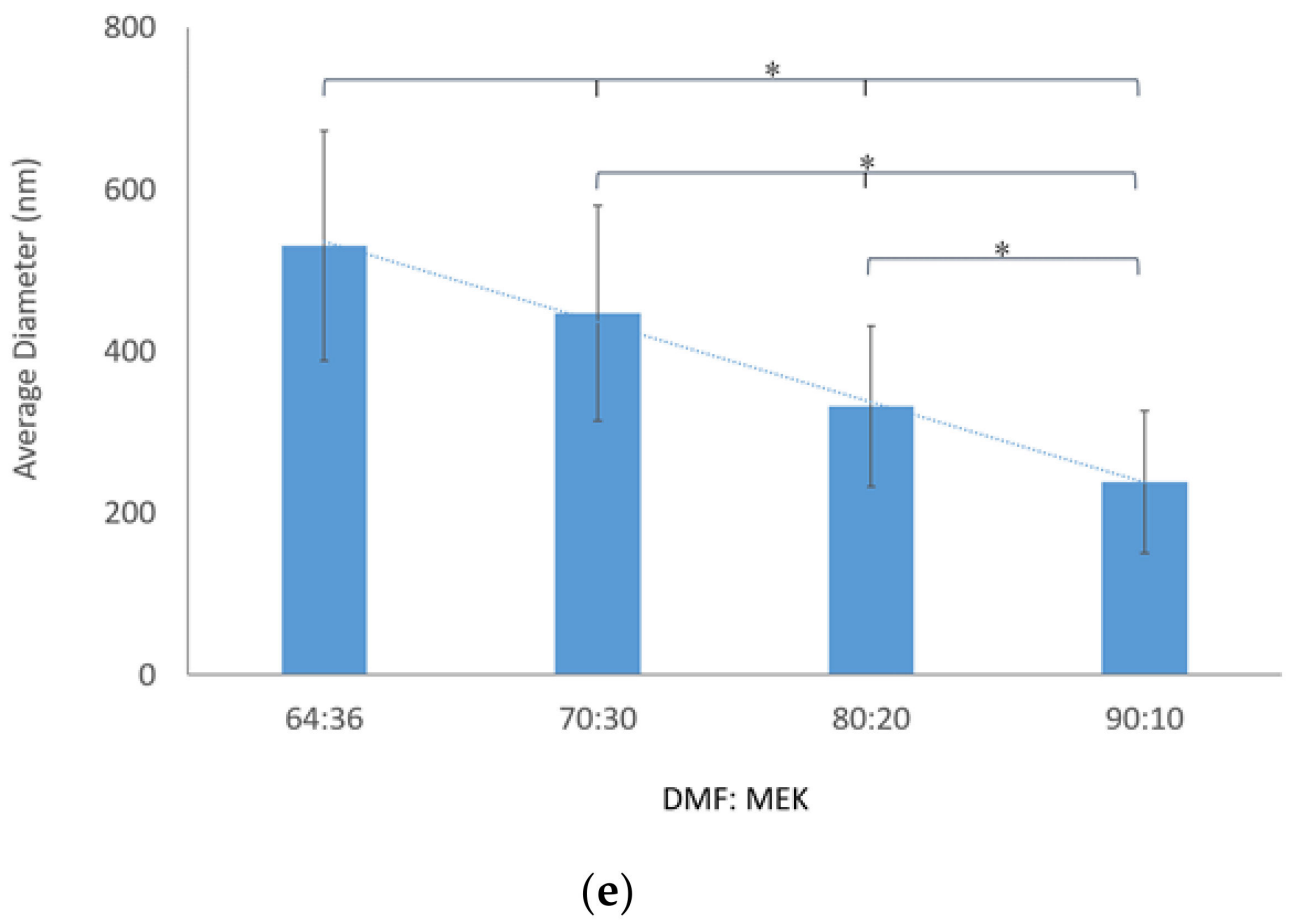
SEM images of PUSX Si01-20 nanofibers under different solvent ratios. (**a**) 64:36, (**b**) 70:30, (**c**) 80:20, and (**d**) 90:10 of DMF:MEK. (Magnification: 2000) (**e**) Average diameters of PUSX Si01-20 nanofibers with different ratios of DMF:MEK. “*” was statistically significant (*p* < 0.05) between each two samples.

Another significant factor that influences the electrospinning process a lot is the selection of solvents. In this study, in order to dissolve PUSX to get an easier electrospinning process, instead of using single solvent DMF, different types of solvents for electrospinning were prepared by using a mixture of DMF and MEK in the weight ratios of 64:36, 70:30, 80:20, and 90:10 in 13% (*w/v*) polymer concentration. PUSX Si01-20 samples were dissolved in the given solvents at room temperature (25 ± 2 °C) with 48 h stirring to obtain homogeneous solutions. Figure 3 presents the fiber morphology variation with a decreasing amount of MEK in the mixture of DMF:MEK; all the four samples showed the fine fiber distributions. When the concentration of MEK was decreased from 30% to 10%, the diameters gradually decreased to 239 nm. This could be related to insufficient resistance of the electrospinning solutions to resist electrical force stretching caused by viscosity, surface tension of the solutions, the hydrophobicity of the polymer, and the boiling point of the solvent [20]. The solvent DMF had a higher boiling point of 152.8 °C than MEK (79.6 °C), and the viscosity and surface tension of the solutions decreased with the ratio of DMF:MEK. It is known that increasing the amount of MEK in the solution can decrease the charge density and electrostatic repulsion due to its lower conductivity (2 × 10^–5^ S/m) than that of DMF (2.5 × 10^–4^ S/m), leading to a trend of stable flow of the solution, stable whipping, stable taylor cone, and improvement of the fiber quality during electrospinning. While increasing the amount of DMF, the stretching of the solution jet was increased as a result of a higher level of charges carried by the solution. The same factor also encouraged the reduction of the fiber diameter [21]. Meanwhile, a higher weight percentage of DMF in the mixed solvent also attributed to the unsmooth surface of nanofibers because of the slow evaporation rate. A unstable electrospinning process appeared with the increase of DMF in the mixed solvents. We could even see the existence of some beads and thinner fibers, as shown in Figure 3b–d. To avoid a rough surface and beads, we chose the ratio of 64:36 (DMF: MEK) to get more uniform fibers. This experiment was also performed on PUSX Si01 and Si05 samples, and similar results were obtained, but are not shown in this paragraph. 

#### 3.1.3. Morphology of PUSX Nanofibers under Optimized Conditions

**Figure 4:**
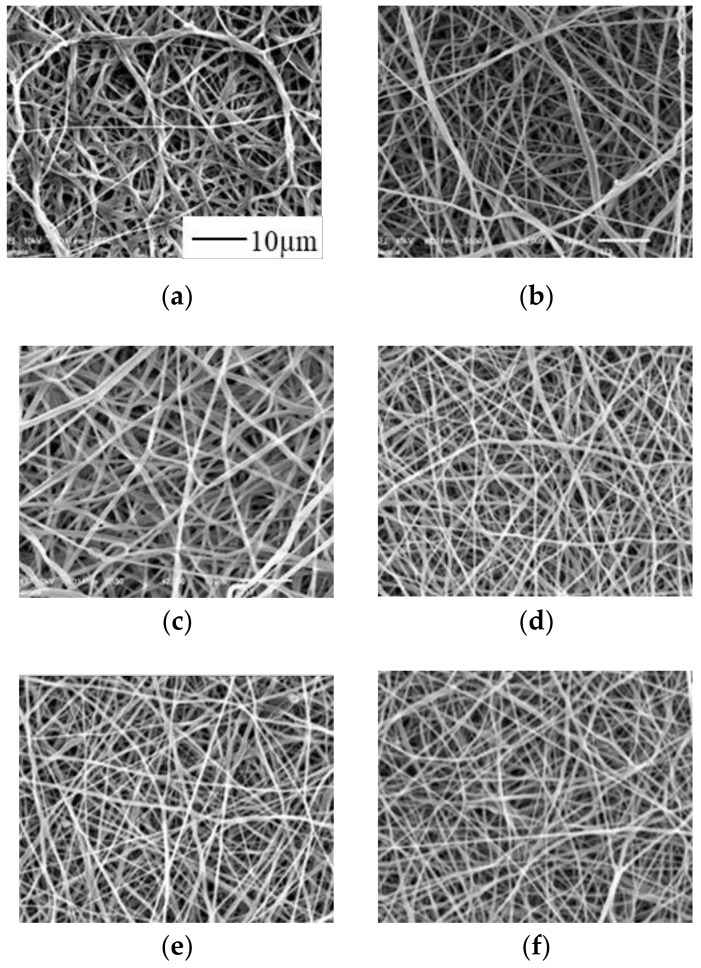

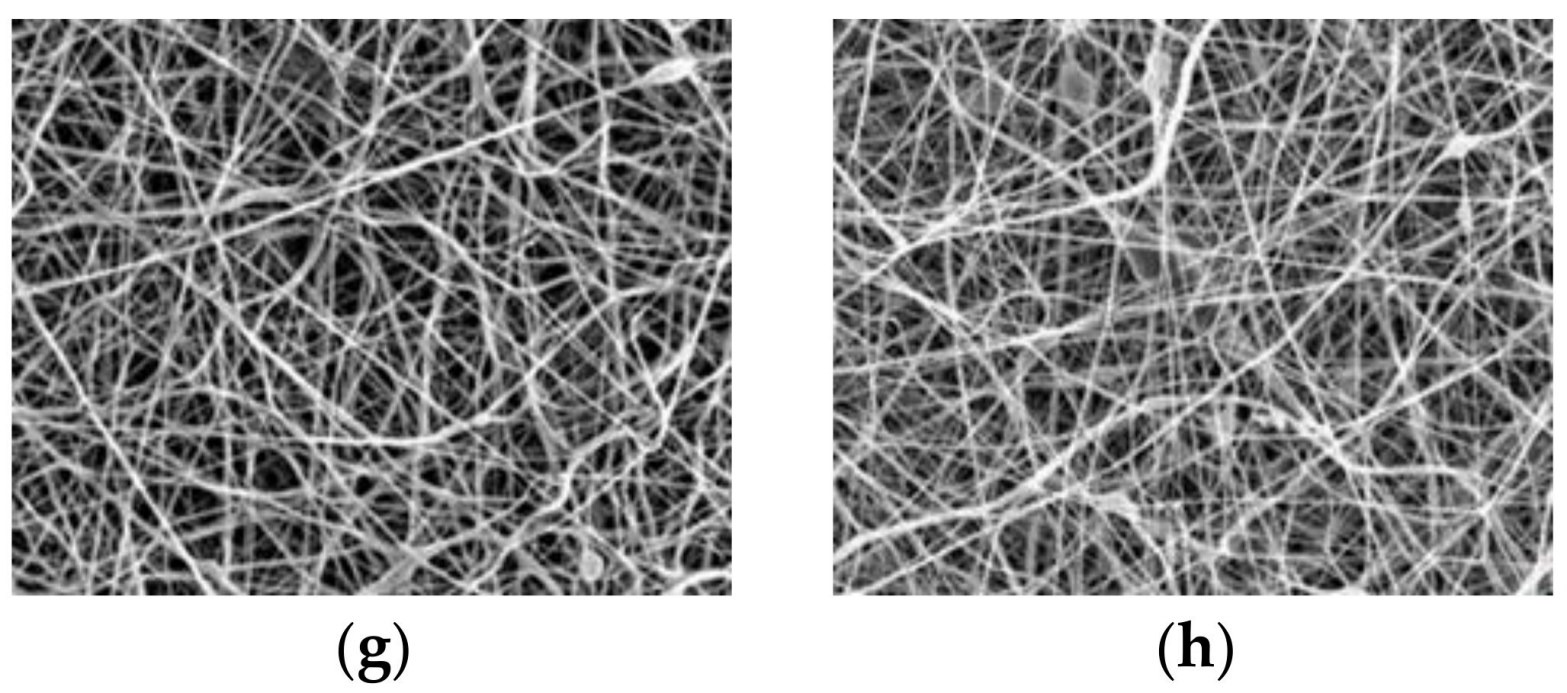
SEM images of block type PUSX nanofibers. (**a**) PU, (**b**) Si01, (**c**) Si02, (**d**) Si03, (**e**) Si04, (**f**) Si01-20, (**g**) Si01-40, and (**h**) Si01-59 under optimized conditions. (Magnification: 2000, DMF:MEK = 64:36).

The fiber morphology of the obtained PUSX nanofibers was studied by means of SEM. Representative SEM images of PU and block type PUSX nanofibers produced with the lab scale device are shown in Figure 4 and Table 3.

It can be seen that the surface morphology of block type PUSX was smooth and continuous, with fiber diameters ranging from 400 nm to 720 nm. The mean diameters of each kind of nanofibers are listed in Table 3. The mean diameters decreased with the increase of both the chain length of silicone and silicone concentration. Table 3 also showed the optimized electrospinning parameters of all the eight kinds of block type PUSX nanofibers. Beadless PU, Si01, Si02, and Si03 PUSX nanofibers could only be obtained by electrospinning solutions with polymer concentrations exceeding 15 wt %. The uniform fine Si04, Si01-20, Si01-40, and Si01-59 PUSX nanofibers could be obtained by electrospinning with polymer concentrations lower than 13 wt %. The diameters and optimized polymer concentrations of nanofibers decreased with an increase of the silicone chain length because of the low surface tension. We supposed that the rather low cohesive force of silicone structure causes the low viscosity by the long silicone chain and high silicone concentration in PUSX. As a result, the optimized polymer concentrations for electrospinning were also adjusted to be low because the surface tension and viscosity highly depend on the structure and concentration of silicone. Meanwhile, low polymer concentrations meant that the solutions contained less polymer so the diameters of nanofibers were decreased. Different silicone chain length PUSX materials were mainly influenced by surface tension while different silicone concentration PUSX materials were mainly influenced by viscosity. This phenomenon can be explained by the component ratio of silicone and polyurethane in PUSX. The ratio of polyurethane to silicone never changed with the silicone chain length while the changes happened in PUSX materials with different silicone concentrations. Compare with PU nanofibers, PUSX nanofibers showed a more uniform surface with a smaller diameter due to the hydrophobicity of the silicone group.

**Figure 5:**
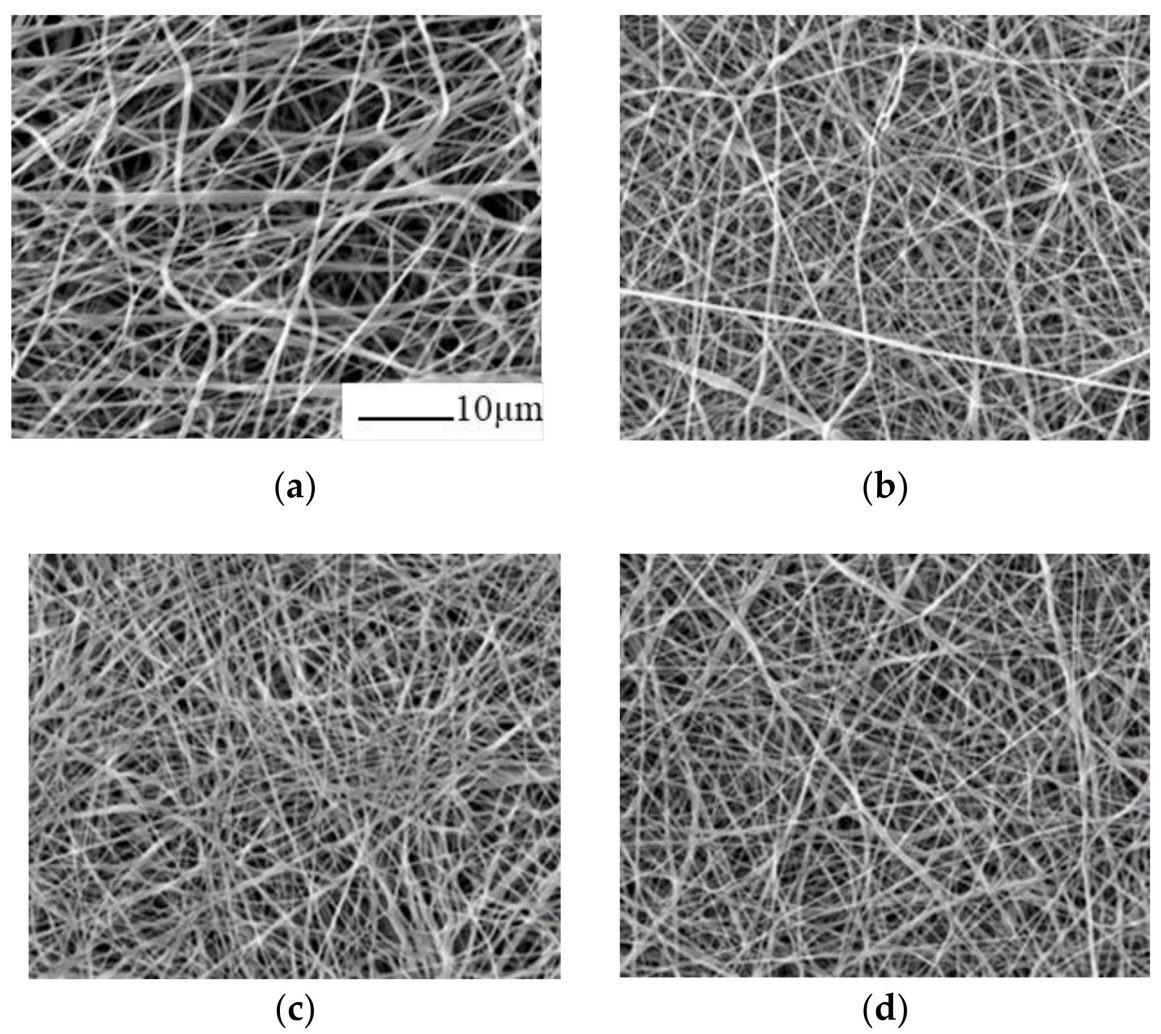
SEM images of graft type PUSX nanofibers. (**a**) Si05, (**b**) Si06, (**c**) Si07, (**d**) Si08 under optimized conditions. (Magnification: 2000, DMF:MEK = 64:36).

Figure 5 and Table 4 show the surface morphologies and the optimized electrospinning parameters for graft PUSX nanofibers. To optimize the electrospinning process of graft type PUSX nanofibers, different polymer concentrations of solutions from 10 wt % to 20 wt % were also tried. It turned out that non-uniform nanofibers with beads could be obtained in the 10 wt % solutions and thicker fibers in the 20 wt % solutions by electrospinning. The most suitable polymer concentrations of graft type PUSX solutions for electrospinning was 15 wt % (same with PU) with the mean diameters ranging from 460 nm to 560 nm. Alike, the surface morphology was fine and continuous. The diameters of nanofibers increased with the polymer concentration and there was no connection found between the silicone chain length and diameters. Compared with block type PUSX, graft type PUSX nanofibers could not show a clear influence of the chain length on electrospinning parameters. This might be because the silicone groups on side chain were not able to influence the characters of electrospinning as much as the silicone groups on the main chain did. It may be concluded that interchain interactions and entanglement of the short side chains were too weak to make differences on the electrospinning jet.

### 3.2. Reproducibility and Upscaling

To investigate the reproducibility of the optimized electrospinning processes using the lab scale set-up and to verify if the optimized conditions can be readily transferred to other devices, the electrospinning process was repeated on a pilot scale device. The processing parameters and various polymer solution concentrations for PUSX Si01 applied on pilot scale electrospinning device are presented in Table 5, and we discussed the possibility of upscale electrospinning under varying nozzle diameters.

In Table 5, SEM images of the electrospun fibers illustrate the good quality (uniform, continuous, and beadless fibers). In addition, the average diameter is also plotted in Table 5 as well as the optimal parameters for upscaling. To commercialize nanofibrous membranes, high-volume production is essential, especially for industrial development in biomedical applications, such as tissue engineering, wound dressings, drug delivery, and for air/liquid filtration and textile applications as well [22]. Considering the growing interest in PUSX for high performance applications, the ability to process PUSX nanofibers in larger quantities was evaluated by a pilot scale set-up, operated with five needles in parallel, and a conveyer belt, which allowed the coating of large areas by continuous electrospinning. Table 5 also shows that the diameter and quality of PUSX fibers obtained by the lab and pilot scale devices were very similar and controllable. To investigate the reproducibility and to verify the previously optimized parameters, we tried the 20 wt % solution and 19 G nozzle needle (ø0.6 mm) at first. It turned out that the diameter was not as small as we obtained on the lab scale set-up. As we know, decreasing the nozzle diameter has the effect of decreasing the fiber diameter, distribution, and productivity [23]. In order to obtain finer fibers, smaller nozzle needles, such as 21 G (ø0.5 mm) and 22 G (ø0.4 mm) were also used to investigate the most suitable parameters on the pilot scale set-up. However, 20 wt % solution was too viscous to form a smooth and stable flow so that a lower concentration became essential. Additionally, the different fiber dimension was influenced by both the concentration and the nozzle diameter.

This experiment demonstrates the feasibility of upscaling the PUSX nanofibers electrospinning process. By controlling the swing speed, rolling speed, and other parameters, we were obtained a nanofibrous sheet with a various and controllable thickness and area. It is worth mentioning that we successfully prepared nanofibers on this pilot scale setup for more than 72 h and obtained nanofiber sheets with an area larger than 23 × 290 cm and a thickness of 0.155–0.175mm. 

### 3.3. Fourier Transform Infrared Spectroscopy (FTIR)

**Figure 6:**
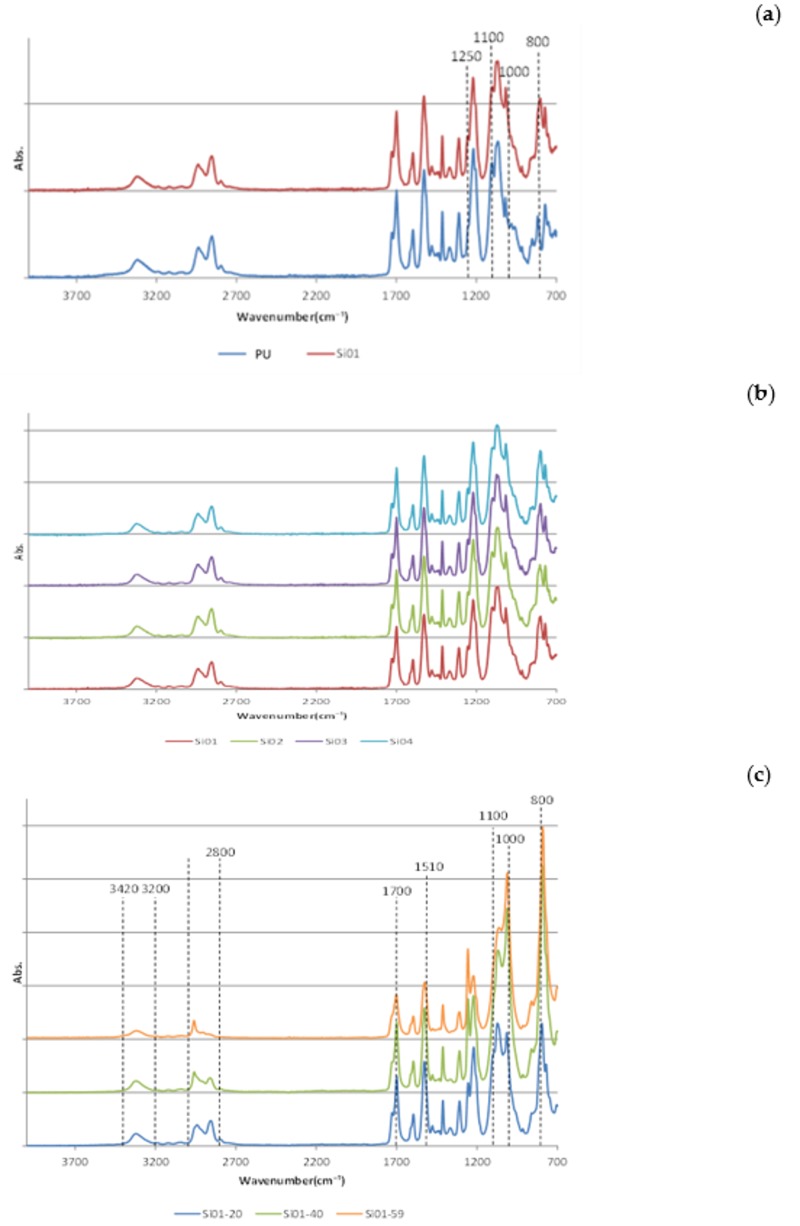

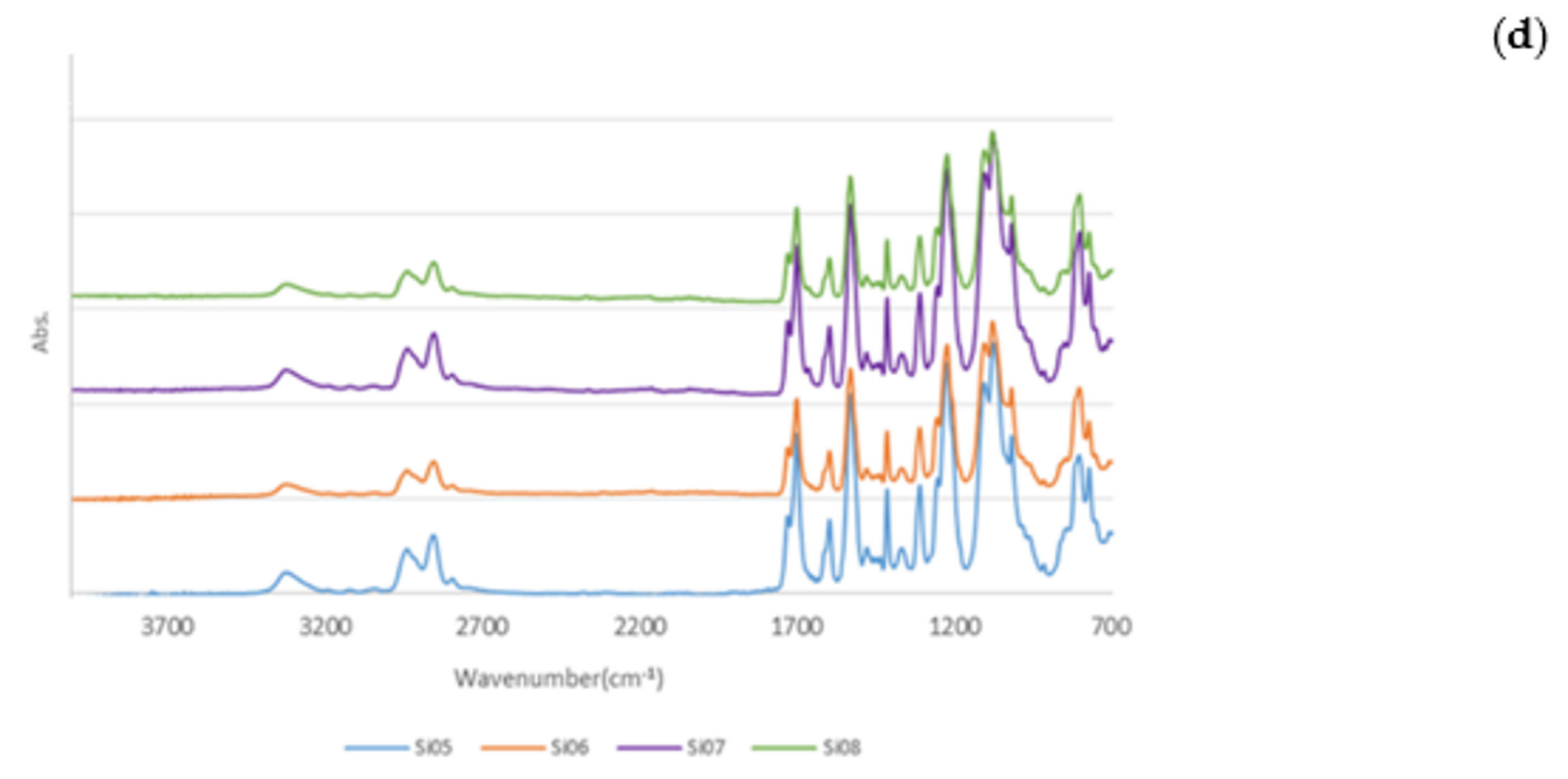
FTIR spectra of PU and PUSX Si01 nanofibers (**a**), block type PUSX nanofibers with various chain lengths (**b**), block type PUSX nanofibers with various silicone concentrations (**c**), graft type PUSX nanofibers with various chain lengths (**d**).

Fourier transform infrared spectroscopy (FTIR) by using ATR for different kinds of PUSX nanofibers was used to observe the chemical structural differences due to the incorporation of a silicone group to the PU polymer chain and electrospinning process. Figure 6 shows the characteristic infrared spectra of the samples.

From Figure 6, it can be seen that the absorption occurred around 3420–3200 cm⁻¹ (NH stretching), 3000-2800 cm⁻¹ (CH₂, CH₃ stretching), 1700 cm⁻¹ (urethane bond), and 1510 cm⁻¹ (amideⅡbond) due to the structure of urethane. Also, the peaks at 1250 cm⁻¹ (the bending of CH in Si-CH₃), 1100–1000 cm⁻¹ (Si-O-C stretching), and 800 cm⁻¹ (Si-C stretching) were characteristic for silicone. The chemical structures of both urethane and silicone in PUSX can be observed from these spectra, which showed that PU was successfully modified by silicone groups. Especially, in Figure 6c, the peaks at 1100–1000 cm⁻¹ (Si-O-Si stretching) and 800 cm⁻¹ (Si-C stretching) were strengthened with the increasing of the silicone concentration in block type PUSX. Moreover, in spectra of PUSX Si01-59, peaks that appeared at 3400–3200 cm⁻¹, 3000–2800 cm⁻¹, 1700 cm⁻¹, and 1510 cm⁻¹ (characteristic bands of urethane) were weakened because of the higher concentration of silicone. In Figure 6b,d, we can see that the peaks were very similar to each other in block type PUSX with different chain lengths and graft type PUSX. This shows that the ratio of PU to silicone in the main chain had the most significant influence on the chemical properties. 

## 4. Conclusions 

In the present study, we successfully prepared 12 kinds of PUSX nanofibers under the optimized conditions and investigated the effects of solvents and solution concentrations on the electrospinnability of PUSX solutions along with the morphological appearance of the as-spun nanofibers and chemical structures were characterized. This is the first time this way of preparing PUSX nanofibers has been suggested during past decades instead of making silicone and PU into composite and then preparing it into nanofibers or films. We reported a new material, which has the advantages of both PU nanofiber and silicone. Silicone groups had greater effects on block type PUSX than graft type. The optimal polymer concentration for electrospinning and the average diameters of the obtained nanofibers decreased with the increase of both the silicone chain length and silicone concentration in block type PUSX nanofibers as well as the decrease of viscosity and surface tension. Graft type PUSX nanofibers did not show a clear influence of the silicone chain length on electrospinning parameters. 

In addition, a study of the reproducibility and feasibility of the electrospinning parameters was performed on a pilot scale electrospinning set-up. On both the lab scale device and pilot scale set-up, PUSX solutions could be successfully electrospun. Hence, the present work demonstrated the great potential of the electrospinning process for the production of PUSX nanofibrous sheets from solutions. The physical properties and biocompatibilities of PUSX nanofibers and films are under investigation and will be discussed in a later manuscript. We believe that PUSX nanofibers might become a new alternative to PU nanofibers or composites in many potential fields. 

## Figures and Tables

**Table 1:** Various parameters (structure, viscosity, molecular weight, and surface tension) of PUSX samples of block type and graft type. (*n* = 3).

	Type	Silicone Concentration (wt %)	Silicone Chain Length (n)	*M_w_* (×10^5^)	*M_n_* (×10^5^)	Viscosity (15 wt %, mPa*s)	Surface Tension (mN/m)
PU	×	0	×	1.48	0.75	700	33.4
Si01	Block	10	20	1.69	0.87	635	24.2
Si02	Block	10	10	1.39	0.73	328	25.5
Si03	Block	10	30	1.59	0.79	378	23.9
Si04	Block	10	50	1.66	0.75	412	24.1
Si01-20	Block	20	20	1.74	0.88	616	23.0
Si01-40	Block	40	20	2.01	1.02	443	22.2
Si01-59	Block	59	20	2.33	1.11	219	21.8
Si05	Graft	10	10	1.56	0.71	520	25.0
Si06	Graft	10	25	1.61	0.70	720	19.1
Si07	Graft	10	30	1.57	0.72	810	23.6
Si08	Graft	10	120	1.62	0.78	820	24.7

**Table 2:**
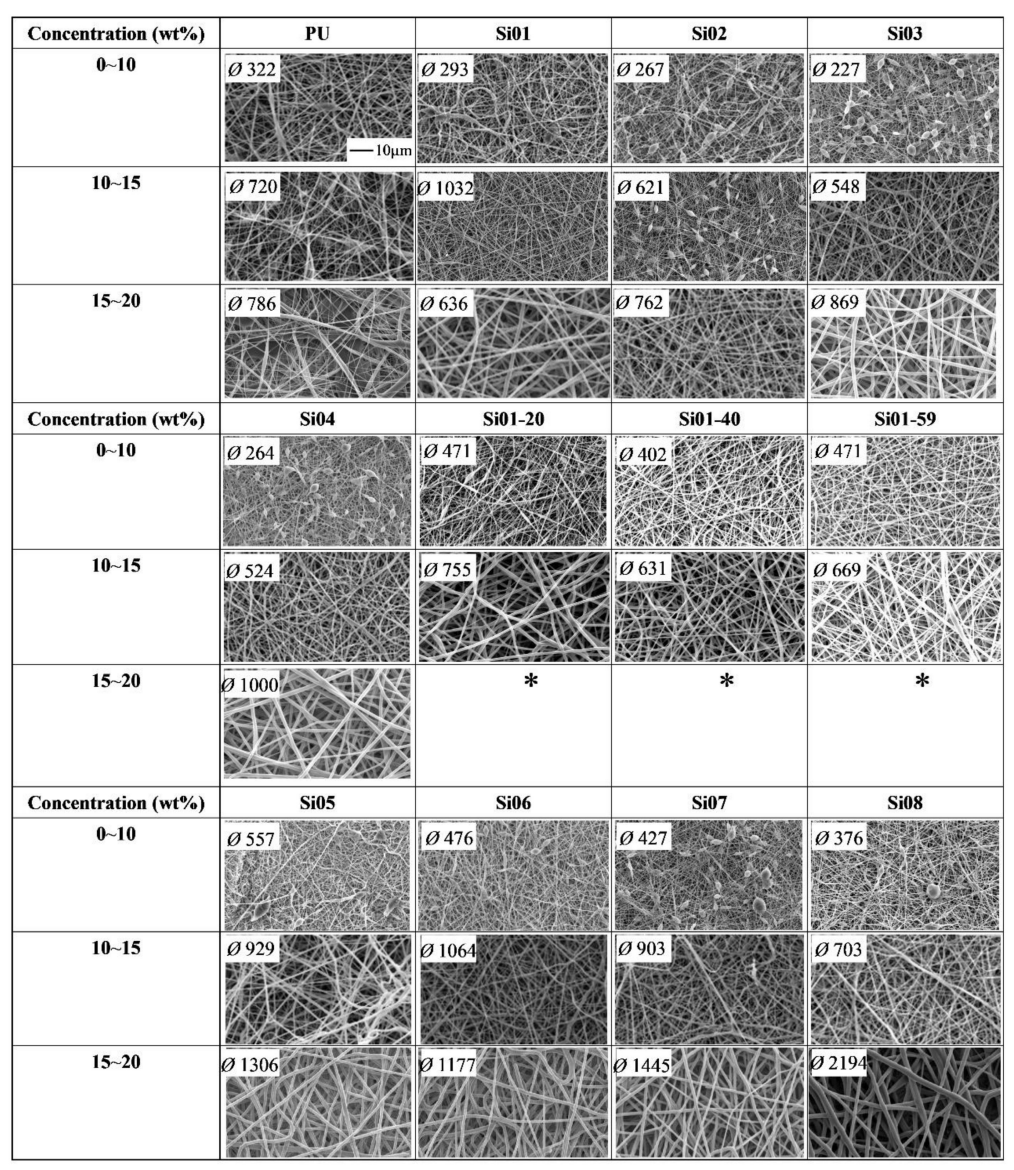
SEM morphologies of the electrospun PUSX nanofibers with various structures and concentrations. (Magnification: 2000, DMF:MEK = 64:36, Ø is the average diameter (nm) of each sample.).

**Table 3:** Average diameters of block type PUSX nanofibers under optimized spinning conditions.

Sample	Polymer Concentration (wt %)	Voltage (kV)	Average Diameter (nm)	SD (nm)
PU	15	20	720	215
Si01	20	20	636	179
Si02	20	20	690	150
Si03	15	20	548	128
Si04	13	20	440	89
Si01-20	13	17	531	142
Si01-40	10	17	402	94
Si01-59	10	17	471	111

**Table 4:** Diameters of graft type PUSX nanofibers under optimized spinning conditions.

Sample	Concentration (wt %)	Voltage (kV)	Average Diameter (nm)	SD (nm)
Si05	15	15	564	142
Si06	15	15	544	124
Si07	15	15	456	117
Si08	15	15	456	129

**Table 5:**
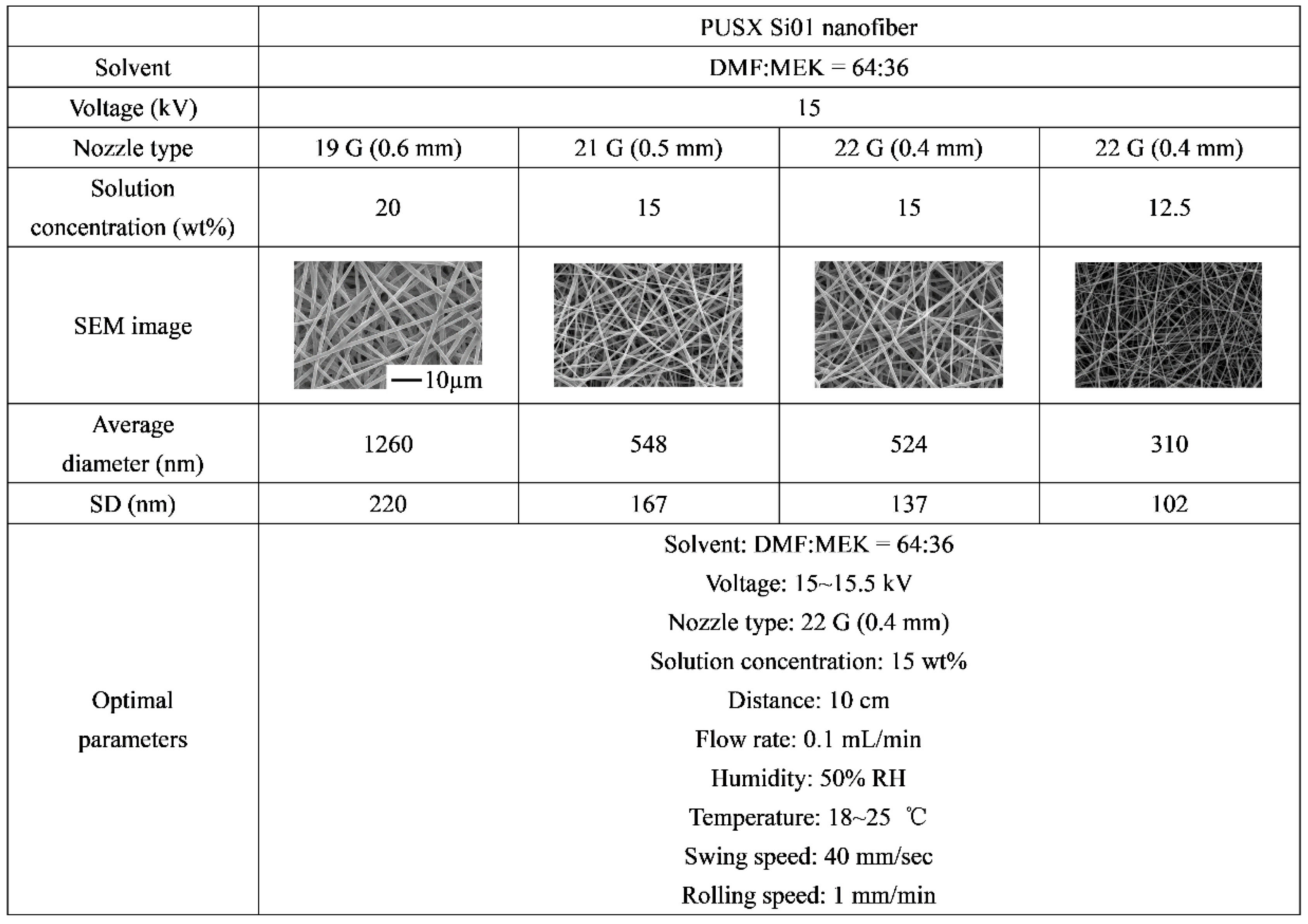
The processing parameters and optimal parameters of PUSX Si01 nanofibers on pilot scale device. (Magnification: 2000).
